# Supplementary material for: Association between oral microbiome and seven types of cancers in East Asian population: a two-sample Mendelian randomization analysis
Source: Front Mol Biosci. 2023 Nov 21;10:1327893. doi: 10.3389/fmolb.2023.1327893 (PMC10702768; doi:10.3389/fmolb.2023.1327893)
Supplement: Supplementary file 1 [file DataSheet1.ZIP › Supplementary Materials/MR plots of saliva/saliva═╝/Breast cancer/pheno.958_to_breast cancer_forest.pdf]

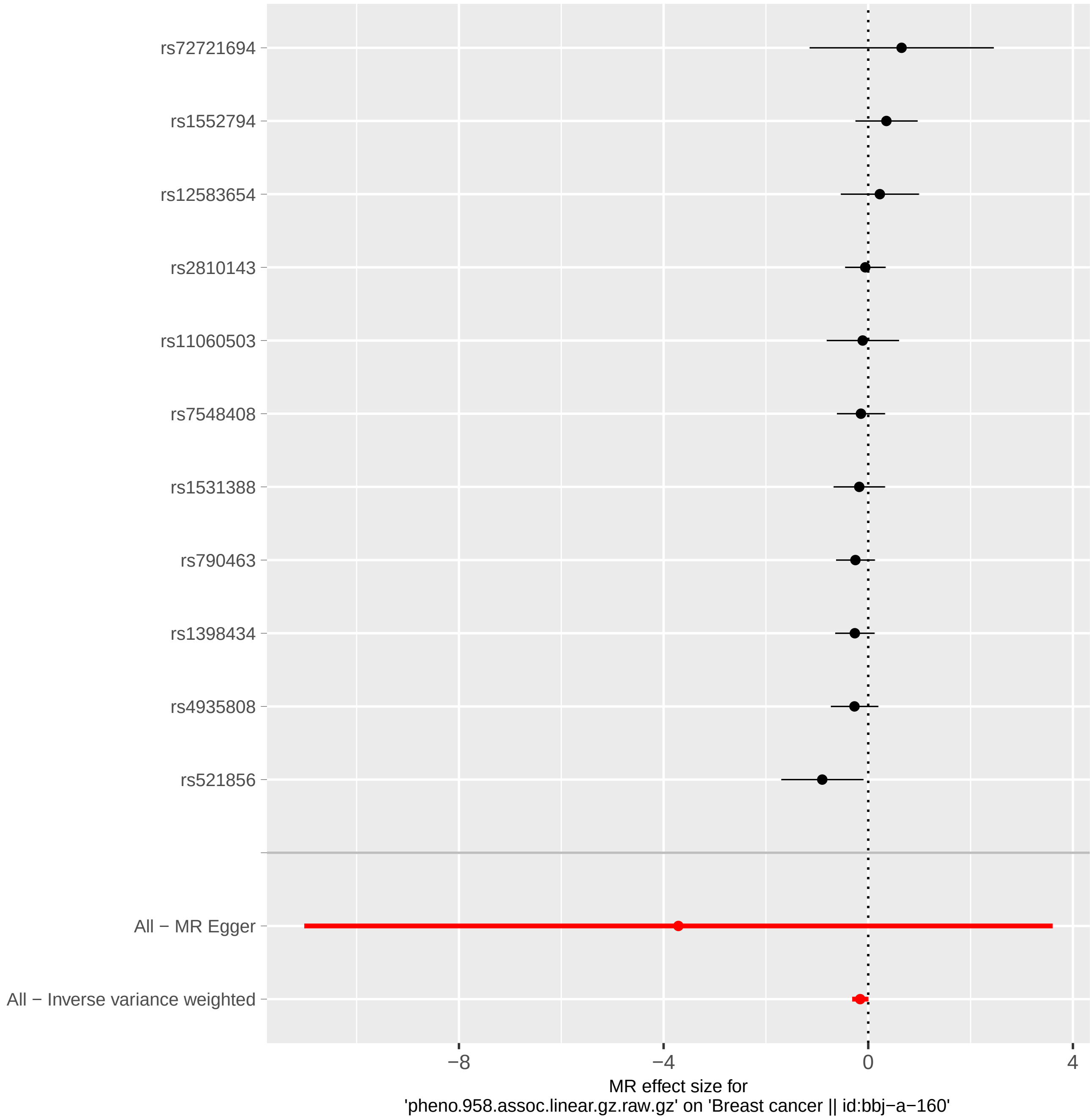

rs72721694
rs1552794
rs12583654
rs2810143
rs11060503
rs7548408
rs1531388
rs790463
rs1398434
rs4935808
rs521856
All − MR Egger
All − Inverse variance weighted
−8
−4
0
4
MR effect size for
'pheno.958.assoc.linear.gz.raw.gz' on 'Breast cancer || id:bbj−a−160'
